# Experimental study of organic enrichment on meiofaunal diversity

**DOI:** 10.1038/s41598-024-60690-7

**Published:** 2024-05-09

**Authors:** Deyaaedin A. Mohammad, Ammar AL-Farga, Mahmoud Sami

**Affiliations:** 1 Department of Marine Science, Faculty of Science, Suez Canal University, Ismailia, Egypt; 2 Department of Biochemistry, Faculty of Science, University of Jeddah, Jeddah, Saudi Arabia

**Keywords:** Organic enrichment, Meiofauna, Nematoda, Experiment, Ecology, Ecology

## Abstract

The organic enrichment effects on the meiofauna and nematofauna were assessed for field sediment and other experimental ones enriched with organic matters conducted in the laboratory for 4 weeks. Also, dissolved oxygen (DO) and pH were monitored for each one. The abundance and diversity of meiofaunal groups and nematofauna varied. Strong significant correlations were found between DO and the studied items. Nematoda was the most abundant group in the field sediment and other experimental ones; their counts increased with the increase in organic enrichments and were dominated by deposit feeders. Amphipoda, Ostracoda and predator/omnivore nematodes disappeared in highly organic-enriched sediments. Changes in DO and organic enrichments might be the more attributable reasons for the alteration of the meiobenthic assemblages. The generic compositions of Nematoda provide a good indicator for environmental alterations.

## Introduction

Meiofauna inhabit intertidal and subtidal soft bottom habitats in water bodies worldwide. Conditions that influence the meiofauna are different from those that affect other components of benthic organisms within the same region. The main significant aspect influencing these animals is possibly the sediment texture, which plays a crucial role in determining the size of interstitial space suitable for dwelling^1^. Coarse sediment grain provides more interstitial space, while fine ones have less interstitial space. Additionally, the granulometry of sediment controls the ability to retain and interchange the water. The exchange of gasses is unrestricted in coarse sediments and reduced in fine-ones^2,3^.

In term of abundance; nematodes, copepods and polychaetes are supposed to be the main meiobenthic groups. Copepoda are usually the second most abundant meiobenthic animals in marine samples^2^. Free living marine nematodes have been used successfully as indicators of biological health and ocean pollution^4–7^ for at least the past 40 years^8^. Before the sample collections, it was generally thought that nematodes were the predominant large group of meiofauna. Additionally, it was believed that the abundance and distribution of meiofauna were strongly influenced by nematodes. For this study, the eulittoral zone of a sandy beach was chosen as the sampling area. This decision was based on the understanding that the eulittoral zone provides the most favorable habitat for meiofauna, as it exhibits greater species diversity compared to other supra/sub-littoral zones, as proposed by Huston's dynamic equilibrium hypothesis^9^.

Armonies and Reise^10^ revealed that there may be an optimal equilibrium among organic supply, oxygen levels, and water retention in the eulittoral zone, which resulted in a high number of taxa per meter interval. This finding was further supported by various studies^11,12^. Due to its unique reproductive strategy, meiofauna is regarded as a significant component in the detrital food chain. It plays a crucial role in both the energy flow of the ecosystem and the ecological assessment of environmental quality. Increasingly, ecologists are utilizing meiobenthic organisms as sensitive indicators to evaluate changes in the environment and community structures^7,13^.

The aim of this study is to evaluate the impact of organic enrichment on meiofaunal community structure and generic corpro of Nematodes.

## Materials and methods

### Field sampling and laboratory procedures

In March 2023, the sandy sediment site (30° 24′ 0.59″ N, 32° 18′ 38.8″ E) was investigated. This site is located on the west side of the Great Bitter Lake, east of Abou-Soultan Power Station and north of Fayed city (Ismailia, Egypt). Agriculture and recreation activities are the main land uses of this region. Meiofauna were sampled from the eulittoral zone (Mid tidal mark) using a hand metal cylindrical corer with an inner diameter of 3.5 cm. The corer device was pushed into the sediment by hand to a depth 10 cm and then pulled out to collect a total volume of 100 cm^3^. Three replicate cores were sampled for the field study. Each sample was transferred into polyethylene bags directly at the site. The sediment samples were preserved in 5% buffered formalin in the field. Water temperature, dissolved oxygen DO, pH and salinity were measured using an YSI multi-parameter device (YSI, Yellow Springs, OH, USA). *Enteromorpha intestinalis* (green algae) was also collected from the adjacent hard substrate.

In the lab, the sediment samples were filtered through a 50-μm screen, and the organisms were collected. The individuals of meiofauna were counted and subsequently sorted into major taxa using a stereo microscope (Prior S2000, magnification 100 ×). Nematodes were examined under a compound microscope (Carl Zeiss 1000 × magnification) and identified to the genus level by pictorial keys^14–16^. According to Wieser^17^, all nematode individuals were categorized to one of the three trophic groups: deposit, epistrate and predators/omnivores feeders.

### Laboratory experimental design

**Figure 1:**
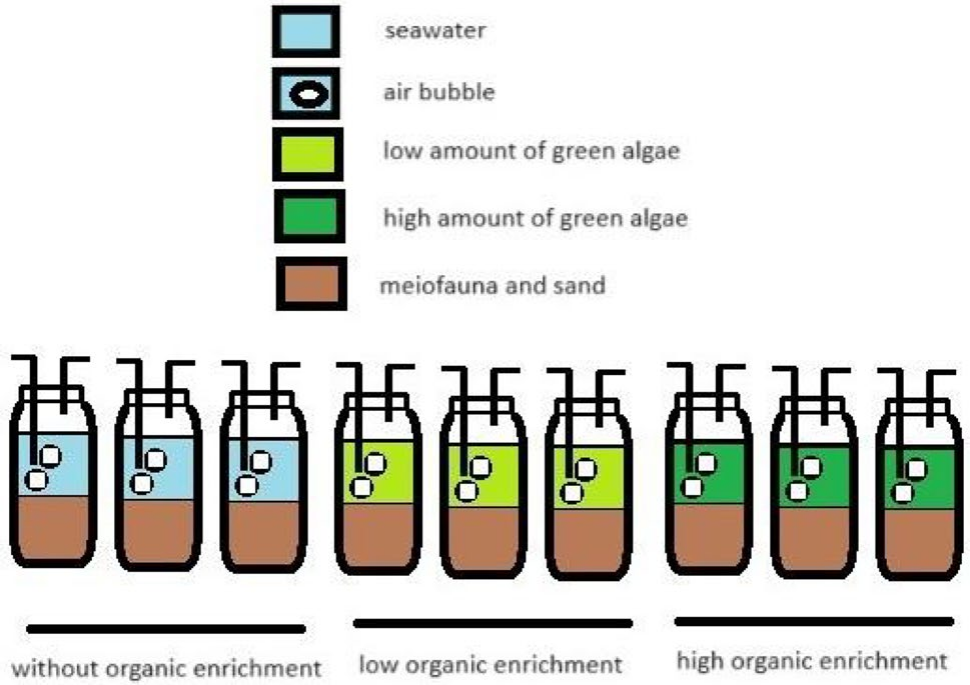
Layout of the laboratory experiment.

Extra sediment samples for experiments were taken from the surface layer (0–10 cm in depth) and immediately transferred to the laboratory. The sediment sample was put into each of the nine jars. These jars were divided into three groups based on their levels of organic enrichment, namely, high-organic enrichment, low-organic enrichment, and control (without enrichment) (Fig. 1). There were three replicates for each enrichment group. In the high-organic enrichment group, 10 g dry weight of green algae (frozen at − 20 °C before treatment, then thawed and dried at 120 °C for 6 h and then powdered with a grinding machine) was added into each of the 3 Jars. On the other hand, 2 g of green algae was added for the low-organic enrichment group. The control group did not receive any amount of algae. So, ratios of added algae among these enrichments were 5: 1:0. The sediment samples were mixed and 100 ml of homogenized sediment was put into each jar. Subsequently, 150 ml of pre-filtered seawater was added to each jar. Finally, each jar was supplied with continuous aeration for 4 weeks. The experiments were carried out under dim light at room temperature to prevent the growth of microalgae. At the end of the experiment, all microcosms were collected, and the samples were preserved in 5% formalin. Also, the pH and dissolved oxygen (DO) levels were measured in each jar.

### Data analysis

The differences in meiofaunal abundance and other major groups among the different groups were examined using a one-way analysis of variance (ANOVA) with a confidence level of 95%. Additionally, Pearson's correlation analysis was conducted to assess the relationships between the total meiofaunal abundance, other major taxa, and the pH and dissolved oxygen (DO). Post-Hoc Tests’ between pH and DO among organic enrichment level were calculated. The above analyses were performed using the statistical software SPSS 18.0 (2002) while PRIMER v6.0 software^18^ used for univariate measures of the diversity indices for meiofauna taxa and nematofauna for field meiofauna samples and other sediments with different organic enrichment levels.

## Results

### Field study

**Figure 2:**
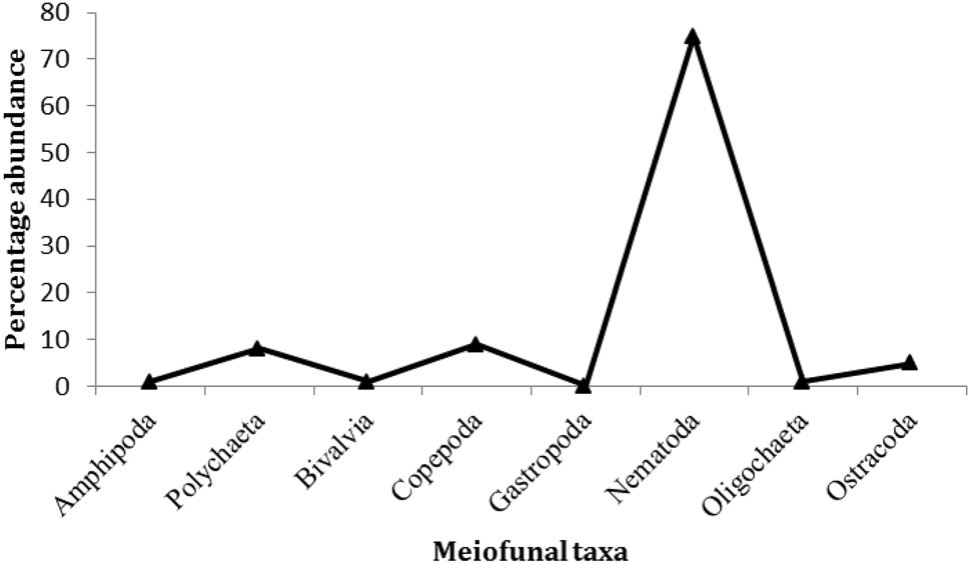
Percentages of the major meiofaunal groups in the studied site.

Water temperature, Salinity, pH and dissolved oxygen values at the study site were 24.3 °C, 38.6‰, 8.2 and 8.7 mg/l, respectively. Meiofaunal community was composed of 8 major taxa (Amphipoda, Polychaeta, Bivalvia, Copepoda, Gastropoda, Nematoda, Oligochaeta and Ostracoda). As observed in Fig. 2, Nematoda (75%) was the most abundant group followed by Copepoda (9%) and Polychaeta (8%). Nematoda recorded the highest average density (170.1 ± 56.3 individual/100 cm^3^) while Gastropoda recorded the lowest one (0.9 ± 1.1 individual/100 cm^3^) (Table 1). For further comparison with the results of experimental study, densities of all taxa at the sampling sites were expresses in number of individual per 100 cm^3^.

**Table 1:** Average individual count/100 cm^3^ for field samples and the experimental study.

	Field meiofauna	Without enrichment	Low organic	High organic	*P* value
Amphipoda	2.3 ± 0.7	2 ± 1	–	–	0.000
Polychaeta	18 ± 5.2	13 ±	8 ± 2.4	17 ± 5.9	0.001
Bivalvia	1.4 ± 0.6	–	–	–	–
Copepoda	20.9 ± 9.3	50.2 ± 13.8	20 ± 5.3	34.5 ± 10.7	004
Gastropoda	0.9 ± 1.1	–	–	–	–
Nematoda	170.1 ± 56.3	187.7 ± 46.5	175.8 ± 16.5	205.5 ± 31.1	0.000
Oligochaeta	1.7 ± 0.3	1.5 ± 1.5	1.9 ± 1.1	4 ± 2	0.005
Ostracoda	11.6 ± 5.6	4.3 ± .8	15.2 ± 1.8	–	0.000
Total meiofauna	227.3 ± 65.7	258.9 ± 32.3	220.7 ± 19.3	261 ± 25.7	

### Laboratory experiments

#### Dissolved oxygen concentration (DO) and pH

**Figure 3:**
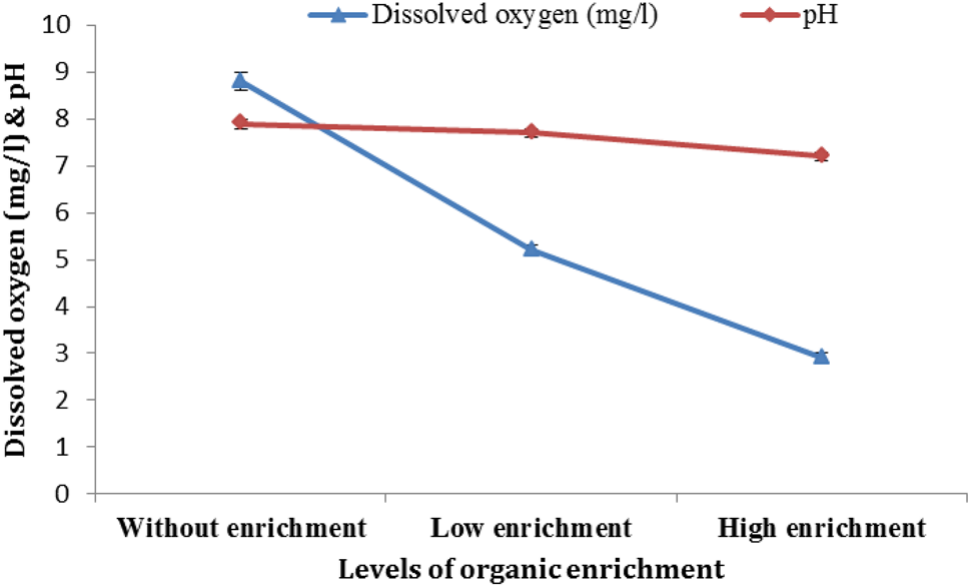
DO and pH average readings in the different level of organic enrichment experiments.

The highest DO and pH values were recorded in without organic enrichment jars (8.8 ± 0.2 mg/l and 7.9 ± 0.1, respectively), while the lowest readings were recorded in high organic enrichment jars (2.9 mg/l ± 0.1 and 7.2 ± 0.1, respectively) (Fig. 3).The differences in DO and pH among the enrichment levels were significant (*P* = 0.000 and *P* = 0.001, respectively). Post-Hoc Tests' Multiple Comparisons revealed significant variations between all enrichment levels in both DO and pH, except for those between sediments without organic enrichment and low organic enrichment ones (*P* = 0.55).

### Meiofaunal abundance

**Figure 4:**
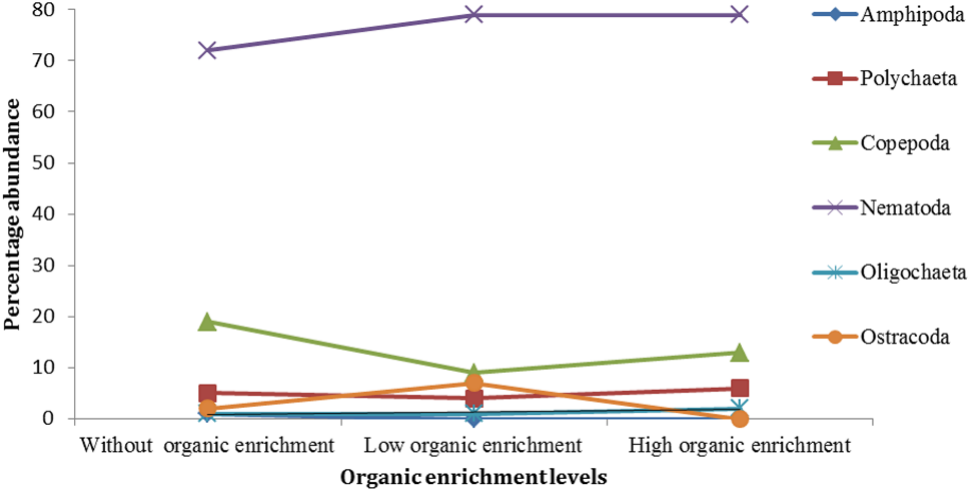
Percentages of major meiofaunal taxa and different organic enrichment levels.

The highest density of total meiofauna was recorded in the high organic enrichment jars (261 ± 25.7 individual/100 cm^3^) while the lowest density was recorded in low organic enrichment jars (220.7 ± 19.3 individual/100 cm^3^) (Table 1). The meiofaunal community was represented by 6 Major taxa in the experimental jars (Amphipoda, Polychaeta, Copepoda, Nematoda, Oligochaeta and Ostracoda). Nematoda was the common taxa in all experiments; their density was increased with the increasing of organic enrichment levels and ranged between 72 and 79% (Fig. 4). Amphipoda, Bivalvia and Gastropoda were absent in organic enrichment jars. (Table 1). One way ANOVA showed a significant variations in taxa density among different enrichments (Table 1).

### Diversity indices of meiofauna

Generally, all diversity indices for meiofaunal assemblages were low. The lowest values of total recorded taxa (S), Shannon–Wiener (H′) and Species Richness (SR) were recorded in the high organic enrichment sediments while the highest diversity indices were recorded in the Field meiofaunal assemblages (Table 2).

**Table 2:** Total recorded taxa (S), total individual count expressed as No. of individual/100 cm^3^ (N), Shannon–Wiener (H′), Species Richness (SR) and Evenness (J′) for both field and different levels of organic enrichment experiments.

	N	S	H′	SR	J′
Field meiofauna	227	8	0.93	1.3	0.45
Without organic enrichment	258	6	0.86	0.89	0.48
Low organic enrichment	220	5	0.79	0.75	0.49
High organic enrichment	261	4	0.72	0.53	0.52

### Nematofauna

**Figure 5:**
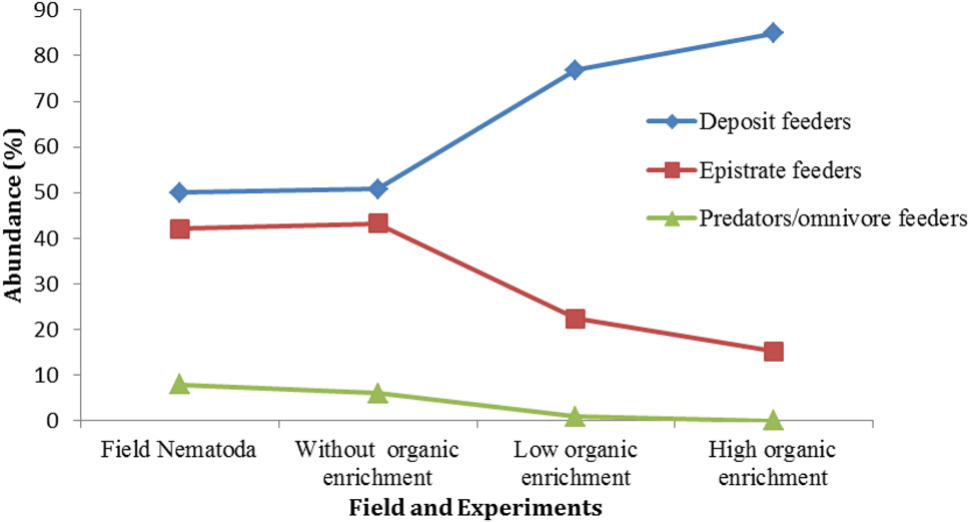
Percentage of nematode feeding groups for field sample and the laboratory experiments.

The highest number of nematode genera (17 genera) was recorded in the field samples, while the least number (7 genera) was recorded in the high organic enrichment sediments. *Daptonema* was the most abundant genus (62.1 ± 11.6 individual/100 cm^3^) in the high organic enrichement sediment. In contrast, 10 genera (*Acantholaimus, Actinonema*,* Dichromadora, Halicoanolaimus*, *Metasphaerolaimus Paralongicyatholaimus*, *Sabatieria, Sphaerolaimus*, *Syringolaimus,* and *Tricoma*) were disappeared in the high level of organic enrichment jars (Table 3). Deposit feeder nematodes were dominated all feeding types and their density ranged from 49% in field samples and 82.6% in high organic enrichement jars. In the other hand, Predator/omnivores was the least feeding type and disappeared in the organic enriched sediments (Fig. 5).

**Table 3:** Average densities of nematode genera and their feeding type for the field and experimental the study.

Genus	Field	Without enrichment	Low enrichment	High enrichment	Feeding type
*Desmosocolex*	14.8 ± 5.6	11.3 ± 4.7	12.6 ± 5.7	19.5 ± 11.6	Deposit feeders
*Halalaimus*	17.5 ± 5.3	25.2 ± 11.7	11 ± 5.4	23.2 ± 15.4	
*Leptolaimus*	9.4 ± 4.7	5.1 ± 2.3	5.6 ± 1.3	12 ± 4.7	
*Tricoma*	6.7 ± 5	4.2 ± 3.6	10 ± 1.3	–	
*Daptonema*	13.5 ± 6.2	11.2 ± 7.5	20.4 ± 8.5	62.1 ± 11.6	
*Sabatieria*	12.2 ± 3.2	7 ± 5	12.2 ± 10.7	–	
*Theristus*	4 ± 1.5	12.6 ± 5.9	15.8 ± 13.9	22 ± 15.3	
*Monhystera*	6.9 ± 2.7	18.2 ± 3.9	27.2 ± 15.3	30.7 ± 17.6	
*Acantholaimus*	27 ± 11.3	22.4 ± 18.3	18.2 ± 7.9	–	Epistrate feeders
*Actinonema*	6.7 ± 3.4	7.8 ± .4	7.1 ± 2.1	–	
*Dichromadora*	5.4 ± 3.1	14 ± 5.5	2.8 ± 2.5	–	
*Microlaimus*	13.5 ± 8.3	16.8 ± 6.9	24.8 ± 3.3	35.4 ± 10.3	
*Syringolaimus*	8.1 ± 0.7	11.2 ± 2.3	5.6 ± 3.7	–	
*Paralongicyatholaimus*	10.8 ± 4.4	9.7 ± 8.3	0.8 ± 1.3	–	
*Halichoanolaimus*	2.7 ± 1.3	–	–	–	Predators/OMNIVORES
*Metasphaerolaimus*	6.8 ± 4.7	4.2 ± 1.7	1.4 ± 0.3	–	
*Sphaerolaimus*	4.1 ± 2	7 ± 1.8	–	–	
Total count	170.1 ± 71.2	187.9 ± 90.3	175.5 ± 69.8	204.8 ± 52.6

**Figure 6:**
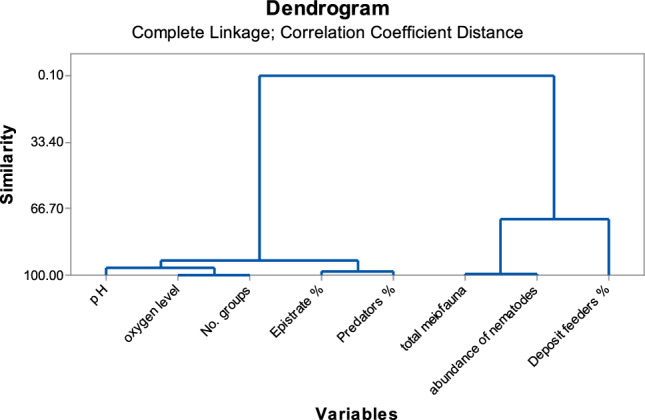
Dendrogram of different studied variables based on correlation coefficient.

Pearson's correlation coefficients between dissolved oxygen, pH, total meiofauna, group abundance, number of recorded taxa and nematode feeding types were calculated for field samples and the experimental enrichment jars. The total recorded taxa showed positively significant strong correlations with DO and pH (*r* = 0.97, *P* = 0.004 and* r* = 0.95, *P* = 0.05; respectively), and also total abundance of meiofauna exhibited the same pattern (*r* = 0.98, *P* = 0.02). Moreover, predator/omnivore nematodes showed a significant strong correlation with DO (*r* = 0.96, *P* = 0.04). The deposit feeding nematodes showed negative strong correlations with the other feeding types. Furthermore the other correlations were not significant. Dendrogram of these correlations revealed two main clusters; the first one constituted of DO, pH, no. of recorded groups, epistrate feeders% and predator/omnivores%. In contrast, deposit feeders, total abundance of meiofauna and nematodes densities constitute the second cluster (Fig. 6).

### Diversity indices of nematofauna

The highest diversity indices of nematofaunal assemblages were recorded for the field sediment while the least ones were recorded for the high organic enrichment ones (Table 4).

**Table 4:** Total individual count expressed as No. of individual/ 100 cm^3^ (N), number of recorded genera (S), Shannon–Wiener (H′), Species Richness (SR) and Evenness (J′) for both field and different levels of organic enrichment experiments.

Parameter	Field	Without enrichments	Low enrichment	High enrichment
N	170	188	175	205
S	17	16	15	7
H′	2.68	2.64	2.45	1.83
SR	3.12	2.86	2.77	1.13
J′	0.95	0.95	0.9	0.93

## Discussion

Three different levels of organic enrichment were conducted to assess the impact of organic enrichment on the meiofaunal community in the laboratory. The design in this study was based on Webb^19^; Armenteros et al.^20^. All nine experimental jars were kept in dark to avoid the growth of photo-autotrophs such as microalgae. The experimental design might to possibly is magnifying the effects of organic enrichment due to the stagnant situations Nevertheless, the experimental setup was considered valuable as it ensured that the essential characteristics of the samples remained consistent between the laboratory experiments and the field study, as noted by Sundbäck et al.^21^.

The organic enrichment of the sediments seemed to control the meiofaunal community structure. Among three different organic content levels, densities of different groups and nematofauna were significantly different. The abundance of total meiofauna and nematode were the highest in high level of organic enrichment sediments, compared with other levels of enrichment. This outcome was deemed satisfactory since it was anticipated that the addition of organic matter would lead to an increase in meiofaunal densities, including both nematodes and other meiofaunal groups.

Wang et al.^2^ and Austen and Widdicombe^22^; established that organic enrichment leads to the rising in meiobenthic densities, which supports the general model of Huston^8^. Also, Pinckney et al.^23^ stated that the eutrophication and organic pollution will lead to increased food supply and a rise in the benthic densities. The abundance and biomass of nematodes are strongly influenced by both food availability and bacterial density. Pascal et al.^24^ discovered, through trophic studies conducted on a tidal mudflat, that nematodes exhibit a preference for microalgae as a food source, in addition to bacterial food. Consequently, a significant increase in meiofaunal densities occurs with high organic enrichment. According to Montagna et al.^13^, Rieper-Kirchner^25^, Gyedu-Ababioa and Baird^26^; Krueger and McSorley^27^ and Nugteren et al.^28^, nematodes are particularly attracted to accumulations of bacteria on plant debris. Furthermore, macrobenthic annelids prefer detritus as their primary food source. Therefore, the introduction of organic enrichment and the subsequent indirect enhancement of bacterial food resources are expected to lead to a general increase in meiofaunal densities, with a specific emphasis on nematodes and annelids.

McLachlan et al.^29^ revealed that, along the coasts of South Africa, meiofaunal abundance was correlated positively with the amount of detritus in the soft bottom habitats. Also, Moreno et al.^30^ found a same correlation for a Mediterranean coast, with low content of organic matter that provides relatively poor meiofauna. In comparisons with the eulittoral zones of the North Sea, with a high content of organic matter, is populated densely^31^.

The concentration of free sulfide should be monitored and analyzed as they may elucidate the meiobenthic fauna in terms of densities and diversities in the present study. Sutherland et al.^32^ found that a high variation of meiofauna was occurred at the presence of high amount of free sulfide. Also, they stated that nematodes exhibited a minor drop in abundance with increasing organic enrichment. In microcosm experiment, Webb^19^ and Armenteros et al.^20^ found different findings from the results in this study. They found high meiofaunal and nematode abundances in low organic level treatments, and the lowest ones were found in high organic level treatments. Moreover, they reported that nematodes and total meiofauna decreased significantly in the high organic enrichment treatments. Furthermore, they suggested that the chemical stressors such as ammonia and hydrogen sulfide derived from reduced conditions in sediments might be important factors affecting the meiobenthic assemblages.

Giere^2^ and Wang et al.^3^ stated that the increase in organic matter will enhance meiofaunal abundance but will also change the community composition and their small scale distribution patterns. Nematoda and Copepoda are responding differently to the environmental alteration. Nematoda are the most resistant group in habitats with low level of oxygen^33–35^. Wang et al.^3^ and Pascal et al.^36^ stated that Harpacticoid Copepoda, Amphipoda and Ostracoda are sensitive to the organic pollution and dissolved oxygen. This status was supported in the current study by investigating the meiofaunal densities relationship to oxygen level and organic content availability. Hypoxia occurred in both organic enrichment levels experiments, which propose the nematode assemblage, might adapt to naturally enriched sediments. The number of recorded taxa and nematode genera were decreased with the increase of organic enrichment. Also Amphipoda and ostracoda were disappeared in the enriched sediments.

In high organic enriched sediment of the present study, 4 groups of meiofauna were found namely; Nematoda (80%), Copepodaes (12%), Polychaeta (6%) and Oligochaeta (2%). Also, Shannon–Wiener and Species Richness value were the least in these high organic enriched sediments. Heip^37^ and Mahmoudi et al.^38^ reported that Shannon–Wiener indices generally have lower values in polluted situations. Herman et al.^39^ and Fraschetti et al.^40^ have reported that taxon diversity tends to be lower under polluted conditions, primarily due to the disappearance of rare taxa such as Ostracoda, Gastrotricha, Halacarida, Hydrozoa, and Tardigrada. In their study along the Belgian coast, Herman et al.^39^ examined meiobenthic communities at 18 stations and found up to seven different higher taxa in sandy stations, while more than 50% of the other stations had only one or two taxa. Amjad and Gray^41^ also observed a decrease in the number of meiofaunal taxa along an organic enrichment gradient. Hodda and Nicholas^42^ discovered a significant correlation between the relative abundance of Oligochaeta and pollution levels, although this group never dominates marine meiobenthic communities.

Also, Keller^43–45^ found that the effect of sewage on the meiobenthic community structures along Marseille coast, France (the heavily polluted coastal regions) were macrofauna are absent, and a relatively poor densities of nematodes, copepods and acari were recorded. All of these findings were supporting the present results.

The nematofauna diversity varied among different levels of organic enrichment in this study; Shannon–Wiener and Species Richness values were exhibited the least values for the sediments of high organic enrichment. Also, dominance of deposit feeders was found due to the increase of organic content. Sabeel and Vanreusel^46^ stated that the alteration in nematode community composition can be attributed to changes in sediment characteristics. Williams^47^, Fleeger et al.^48^ and Mohammad^49^ found that good relation between the distribution of nematode species directly to pore-space, while more recent studies suggest that the shift in size class is more likely the response of organisms to other sediment-related physic–chemical parameters such as oxygen concentration^32,50,51^, abundance of microalgal biomass^52^ and sediment disturbance^53^. Small nematodes such as *Daptonema* and* Microlaimus* are considered as colonizers i.e. small individuals that show rapid growth, and early reproduction^46,53^. This is in accordance with results of the current study where *Daptonema* and *Microlaimus* were showed increase of their abundances with increasing organic enrichment levels. These results are appearing shift in dominance of large bodied species with low turnover rates toward dominance of small-bodied species with high turnover rates (as opportunist or r-strategist species).

In the current study, predator nematode genera were absent in the organic enriched sediments and displaying a positively strong significant correlation with oxygen level. This finding is consistent with Vitiello and Aissa^54^ who studied the nematode communities in polluted lagoon of Tunisia and found only three nematode genera were characteristic of the organically polluted communities*.* Also, they found that the absence of the predator nematode in this area while they accounted for about 15% of the communities in the unpolluted region. Also, Giere^2^ reported that the oxygen requirements of nematode was the highest for predator ones. This status might be elucidating the absence of these genera in the high organic enriched sediment in the present study.

## Conclusion

Changes in some water quality parameters can affect the meiofauna/nematofauna assemblages due to the organic enrichment of sediment. Its community structures considered good indicators for environmental impacts and healthiness. Further detail studies (the biomass of nematodes, concentrations of free sulfide and unionized ammonia) on the composition of nematode communities in the organic-enrichment microcosm experiments are needed. Also, longer experiment period (more than four weeks) perhaps could be significantly useful for future works.

## Data Availability

The datasets used and/or analyzed during the current study available from the corresponding author on reasonable request.
